# Clinical value of combined ^18^F-FDG and ^18^F-P3BZA imaging in the diagnosis of melanoma

**DOI:** 10.3389/fmed.2025.1571929

**Published:** 2025-05-09

**Authors:** Rongchen An, Tian Xiang, Feng He, Xiaowei Ma, Yunhua Wang

**Affiliations:** Department of Nuclear Medicine, The Second Xiangya Hospital, Central South University, Changsha, China

**Keywords:** ^18^F-FDG, ^18^F-P3BZA, PET/CT, melanoma, melanin

## Abstract

**Objective:**

This study aims to explore the clinical value of the combination of ^18^F fluorodeoxyglucose (^18^F-FDG) and N-[2-(dimethylamino) ethyl]-^18^F-5-fluoropicolinamide (^18^F-P3BZA) positron emission tomography/computed tomography (PET/CT) in melanoma patients.

**Methods:**

A retrospective study was conducted on 19 melanoma patients who underwent ^18^F-FDG and ^18^F-P3BZA PET/CT scans at the Nuclear Medicine Department/PET Imaging Center of the Second Xiangya Hospital, Central South University, from March 2022 to March 2024. The diagnostic efficacy of ^18^F-FDG, ^18^F-P3BZA, and the combination of both for melanoma was compared, and the value of combined imaging for TNM staging and clinical treatment decision-making in melanoma patients was discussed.

**Results:**

The sensitivity of ^18^F-P3BZA in diagnosing primary lesions of melanoma, all metastases, sentinel lymph node metastases (SLNM), distant lymph node metastases (DLNM), and bone metastases (BM) was 100% (12/12), 71.4% (40/56), 72.4% (21/29), 66.7% (14/21), and 83.3% (5/6), respectively. The corresponding values for ^18^F-FDG were 91.7% (11/12), 91.1% (51/56), 86.2% (25/29), 95.2% (20/21), and 100% (6/6), respectively. Combined imaging showed a higher sensitivity in diagnosing SLNM, DLNM, and all metastases than ^18^F-P3BZA (*χ*^2^ = 7.105, *p* = 0.004; *χ*^2^ = 3.860，*p* = 0.045; *χ*^2^ = 15.604; *p* < 0.001). In addition, the specificity of ^18^F-FDG in diagnosing all metastases, SLNM, DLNM, and BM, was 50.0, 69.2, 56.3, and 100%, respectively, and the corresponding values for ^18^F-P3BZA were 81.8, 100, 75.0, and 100%, respectively. Combined imaging improved N and M staging in 31.6% (6/19) of melanoma patients and changed clinical treatment decisions in 26.3% (5/19) of melanoma patients.

**Conclusion:**

The specificity of ^18^F-FDG alone in diagnosing melanoma is low, but it can be combined effectively with ^18^F-P3BZA. The combination of ^18^F-FDG and ^18^F-P3BZA PET/CT can further improve the detection efficiency of lesions, TNM staging, and clinical treatment decisions.

## Introduction

Melanoma is one of the most aggressive types of cancer, and its incidence continues to rise globally (1). It usually occurs on the skin of the extremities, with its incidence peaking at the age of 65 years; however, it can affect individuals of any age (2). The American Joint Committee on Cancer (AJCC) points out that the 5-year survival rate for early-stage patients after surgery is higher than 95% and that the incidence of distant metastasis is less than 10% (3, 4).

The diagnosis of skin melanoma is performed through physical examination and local biopsy by clinical physicians, whereas for its clinical staging and treatment evaluation, imaging methods are required. However, on the one hand, conventional imaging generally provides morphological information but has low specificity; on the other hand, due to its single-site imaging, its staging value is relatively low (5, 6).

Positron emission tomography/computed tomography (PET/CT) has been widely used for staging, post-treatment restaging, and evaluating the efficacy of various solid malignant tumors in humans (7–10). ^18^F fluorodeoxyglucose (^18^F-FDG) PET/CT has changed the diagnostic approach of melanoma and has become the most clinically valuable method for clinical staging and follow-up treatment of melanoma (1, 11, 12). However, ^18^F-FDG has some limitations, primarily due to its low specificity. In addition, the high uptake of brain tissue during ^18^F-FDG PET/CT imaging may affect the detection of brain metastases (13, 14).

In recent years, various novel molecular probes for melanoma have been developed, including benzamide (BZA) and its derivatives, antibodies, receptors, very late antigen-4 (VLA-4), and metabolic glutamate receptor-1 (mGluR1) (1, 15–19). Among these, BZA derivatives have been the most extensively investigated. Due to the presence of melanin in more than 90% of melanoma cells, targeting melanoma lesions by targeting melanin is theoretically possible. BZA derivatives are polycyclic aromatic compounds and can specifically bind to melanin in cells (20, 21). Therefore, melanoma lesions can be targeted by labeling BZA derivatives with radioactive isotopes.

In 2013, Liu et al. (22) designed and synthesized several ^18^F-labeled BZA analogs as positron probes for targeting melanoma lesions in B16F10 tumor-bearing mice. They found that N-[2-(dimethylamino) ethyl]-^18^F-5-fluoropicolinamide (^18^F-P3BZA) had a highly specific melanin-binding ability and the highest target-to-background uptake ratio. In 2019, Ma et al. (23) utilized ^18^F-P3BZA PET/CT for the diagnosis of melanoma and found that its imaging effect was superior to that of ^18^F-FDG PET/CT. This finding confirms that ^18^F-P3BZA has the potential for clinical application. However, this study included only five cases of melanoma and did not analyze the detection rate of the lesions, let alone clarify their exact clinical application value.

The present study included 19 patients with melanoma and compared the clinical application value of ^18^F-P3BZA PET/CT, ^18^F-FDG PET/CT, and the combination of both. This study adhered to the principles of the Declaration of Helsinki and was approved by the Ethics Committee of Xiangya Second Hospital, Central South University (ethics approval number: 2021–165). All participants provided their signed informed consent.

## Patients and methods

### Patients

We conducted a retrospective analysis of melanoma patients who underwent ^18^F-FDG and ^18^F-P3BZA PET/CT scans at the Nuclear Medicine Department/PET Imaging Center of the Second Xiangya Hospital, Central South University, from March 2022 to March 2024. Clinical data and PET/CT scan data of the patients were collected, and it was ensured that the interval between two PET/CT scans did not exceed 7 days.

Inclusion criteria were as follows: (1) those diagnosed with melanoma by pathological findings; (2) those with access to clinical and pathological data; (3) those who accepted to undergo ^18^F-FDG and ^18^F-P3BZA PET/CT scans with good image quality; and (4) those with complete follow-up information.

Exclusion criteria were as follows: (1) those who had incomplete clinical data or were lost to follow-up; (2) having other malignant tumors or serious systemic diseases; and (3) those with contraindications for radionuclide scanning.

Based on these criteria, 19 patients were included in this study.

### Synthesis and quality control of ^18^F-P3BZA and ^18^F-FDG

^18^F-P3BZA: ^18^F-P3BZA was generated using a cyclotron at the Nuclear Medicine Department/PET Imaging Center of the Second Xiangya Hospital, Central South University (Siemens, Germany). ^18^F-P3BZA was synthesized using the AllInOne™ synthesizer (TRASIS, Belgium) based on previous research methods (23). Both the radiochemical purity and labeling rate of ^18^F-P3BZA were higher than 95%.

^18^F-FDG: ^18^F-FDG was synthesized using the Explora FDG4 and Explora GN chemical synthesis modules, and its radiochemical purity and labeling rate were both higher than 95%.

For PET/CT, uMI780 PET/CT (Shanghai United Imaging Healthcare Co., Ltd.) was used.

### ^18^F-FDG PET/CT image acquisition

The ^18^F-FDG injection was administered at a dosage of 3.5 MBq/kg. Before administering the injection, patients were asked to fast for at least 6 h, and it was ensured that their blood sugar level did not exceed 11.1 mmol/L. Patients were subjected to scanning 60 min after injection, with the scan area being from the top of the skull to the sole of the plantar. First, CT and PET scans of the body (neck to the plantar) were performed with the following parameters: voltage: 120 kV; radiation dose: 120 mAs; matrix: 600 × 600; and layer thickness: 3.75 mm. PET acquisition was performed in 3D, with a speed of 2 min per bed for a total of 5–9 beds. Then, separate head CT and PET acquisitions were performed, with a head PET acquisition speed of 2 min per bed for one bed. The collected data were reconstructed using iterative methods and transferred to an MMWP image post-processing workstation.

### ^18^F-P3BZA PET/CT image acquisition

The ^18^F-P3BZA injection was administered at a dosage of 2.5 MBq/kg (lower than Ma et al.’s study dose for radiation safety issues), and scanning was performed 60 min after the injection (our team’s previous research confirmed the highest target background uptake ratio at 60 min). Patients were instructed to drink approximately 200–500 mL of water 10 min before scanning to dilute and excrete the radioactive accumulation of ^18^F-P3BZA in the stomach. The scanning range was from the top of the skull to the plantar, and the patients were asked to remain stationary on the examination bed throughout the entire process. First, a low-dose CT scan was performed with the following parameters: voltage: 120 kV; radiation dose: 120 mAs; matrix: 600 × 600; and layer thickness: 3.75 mm. Then, another PET scan was performed, with 3D PET acquisition at a speed of 2 min per bed for a total of 6–10 beds. The collected data were iteratively reconstructed to obtain whole-body CT images, PET images, and PET/CT fusion images. Then, the reconstructed data were transferred to the MMWP image post-processing workstation.

### Image analysis

Two senior nuclear medicine physicians who were involved in PET/CT diagnosis jointly reviewed the PET/CT images. In case of disagreement, the final decision was made through mutual consultation and agreement between the two physicians. The images were primarily analyzed using visual and quantitative methods.

#### ^18^F-FDG pet/CT

Visual analysis: Except for lesions in the brain and kidneys, any lesions characterized by localized skin thickening, nodules, or masses, along with an increased radioactive uptake or significantly higher radioactive uptake compared with the surrounding normal tissues, were considered primary lesions. For a lymph node (LN) in the drainage pathway of the melanoma, if its volume or radioactive uptake was found to increase, it was considered a metastasis. If there was a bone change on the whole-body scan with increased local radioactive uptake, it was considered a bone metastasis (BM).

Quantitative analysis: For a primary lesion, in addition to monitoring its morphological changes, if its maximum standardized uptake value (SUVmax) was higher than 2.5, it was considered a primary melanoma lesion (24, 25). For an LN in the drainage pathway of the melanoma, if the short diameter was higher than 10 mm or its SUVmax was higher than 2.5, it was considered a metastasis. If there was a change in bone density or the SUVmax was higher than 2.5 in the whole-body scan, it was considered a BM.

#### ^18^F-P3BZA pet/CT

Visual analysis: In ^18^F-P3BZA PET/CT images, except for lesions in the kidneys, liver, gallbladder, spleen, and stomach, any lesions characterized by local skin thickening, nodules, or masses, along with an increased radioactive uptake or significantly higher radioactive uptake than the surrounding normal tissues, were considered primary lesions. If the volume or radioactive uptake of an LN in the drainage pathway of the melanoma was found to increase, it was considered a metastasis. If a bone change was observed on the whole-body scan along with increased local radioactive uptake, it was considered a BM.

Quantitative analysis: If the SUVmax of a primary lesion was higher than 2.5, in addition to showing morphological changes, it was considered a primary melanoma lesion. If the short diameter of an LN in the drainage pathway of the melanoma was higher than 10 mm or its SUVmax was higher than 2.5, it was considered a metastasis. If a change in bone density was observed on the whole-body scan and the SUVmax was higher than 2.5, it was considered a BM.

Clinical TNM staging of melanoma patients was recommended according to the latest AJCC guidelines (26).

### Follow-up

For each patient, follow-up began at the end of their scan and continued for at least 1 month. Among the 19 melanoma patients, 12 were newly diagnosed, while 7 received postoperative care after their surgery. All primary lesions were validated using pathological results. Metastases were validated through histopathological, clinical, and imaging follow-up (including ultrasound, CT, MRI, and PET/CT) results. If a lesion was found to be significantly enlarged or had a significantly increased metabolism during follow-up, or if a lesion was found to be significantly shrunken or had a significantly decreased metabolism after drug treatment, it was considered a metastasis.

### Statistical analysis

The diagnostic consistency between two nuclear medicine physicians was evaluated using the Kappa test. IBS SPSS 26.0 and Origin 2023 were used for statistical analysis and image plotting. Count data were expressed as mean ± standard deviation (x ± s), frequency, and percentage (%). In addition, the *t*-test was performed to compare the differences in semi-quantitative parameters between ^18^F-FDG and ^18^F-P3BZA, and the Chi-squared test was utilized to compare the differences in diagnostic efficacy between ^18^F-FDG, ^18^F-P3BZA, and combined imaging. A *p*-value of less than 0.05 was considered statistically significant.

## Results

### Study population

Of the 19 melanoma patients, 12 were initially diagnosed, and 7 underwent postoperative follow-up. Partial clinical data and PET/CT scan results of the 19 patients are presented in Table 1.

**Table 1:** Clinical and PET/CT scan data of 19 melanoma patients.

Patient number	Sex	Age(y)	Height(cm)	Weight(kg)	Primary site	^18^F-FDG dose (MBq)	^18^F-P3BZA dose (MBq)	Confirmed metastases^*^	TNM
1	F	45	156	61	Right external genitalia	248	200	LNM	TxN2M0
2	F	47	156	65	Right neck	274	222	LNM	TxN1M0
3	F	73	170	59	Right plantar	259	207	LNM	TxN0M1a
4	M	35	168	67	Left plantar	289	204	-	T3bN0M0
5	M	76	170	77	Left plantar	300	248	LNM	TxN0M1a
6	F	36	156	73	Right calf	270	204	LNM	TxN2M0
7	F	74	145	56	Right plantar	241	244	LNM, BM	T3bN3M1c
8	F	68	153	48	Right thigh	174	222	-	TxN0M0
9	M	68	163	65	Right finger	259	185	LNM	TxN2M0
10	M	51	166	57	Left plantar	211	226	-	T2aN0M0
11	M	62	164	65	Right toe	259	200	LNM	TxN2M0
12	M	44	175	97	Right plantar	359	259	-	TxN0M0
13	M	72	161	52	Left finger	222	211	LNM	TxN2M0
14	F	39	153	51	Right eye	226	189	-	/
15	F	65	151	69	Left eye	344	81	-	/
16	F	70	150	65	Right plantar	233	130	-	TxN0M0
17	M	55	176	80	Right plantar	167	130	-	TxN0M0
18	M	33	168	72	Left anterior chest	274	215	-	TxN0M0
19	F	67	144	48	Right toe	178	192	LNM	TxN3M0

Histopathological results and follow-up confirmed 56 metastases in 11 patients (49 lymph node metastases (LNM) in 10 patients and 1 LNM and 6 BM in 1 patient) of the 50 LNM, 29 were sentinel lymph node metastases (SLNM).

### Distribution of ^18^F-P3BZA in melanoma patients

The distribution of ^18^F-P3BZA in important organs and tissues of 19 patients with melanoma is presented in Table 2.

**Table 2:** Distribution of ^18^F-P3BZA in important organs and tissues of 19 melanoma patients.

Sites	Uptake level	SUVmax	SUVmean
Stomach	High, diffuse	6.4 ± 4.1	4.1 ± 2.8
Liver	Medium/high, diffuse	6.1 ± 2.0	4.4 ± 1.7
Gallbladder	High, diffuse	10.0 ± 5.7	7.3 ± 4.0
Spleen	Medium/high, diffuse	4.0 ± 1.2	2.8 ± 0.9
Mediastinal blood pool	Low, diffuse	1.2 ± 0.3	0.7 ± 0.2
Lung	Low, diffuse	1.2 ± 0.2	0.8 ± 0.1
Muscle	Low, diffuse	1.5 ± 0.3	0.9 ± 0.2
Eye (choroid)	Medium/high, diffuse	1.9 ± 0.6	1.3 ± 0.4
Bone	Low/medium, diffuse	3.6 ± 0.9	2.3 ± 0.5

SUVmax, maximum standardized uptake value; SUVmean, mean standardized uptake value.

### ^18^F-P3BZA PET/CT scan results

**Figure 1:**
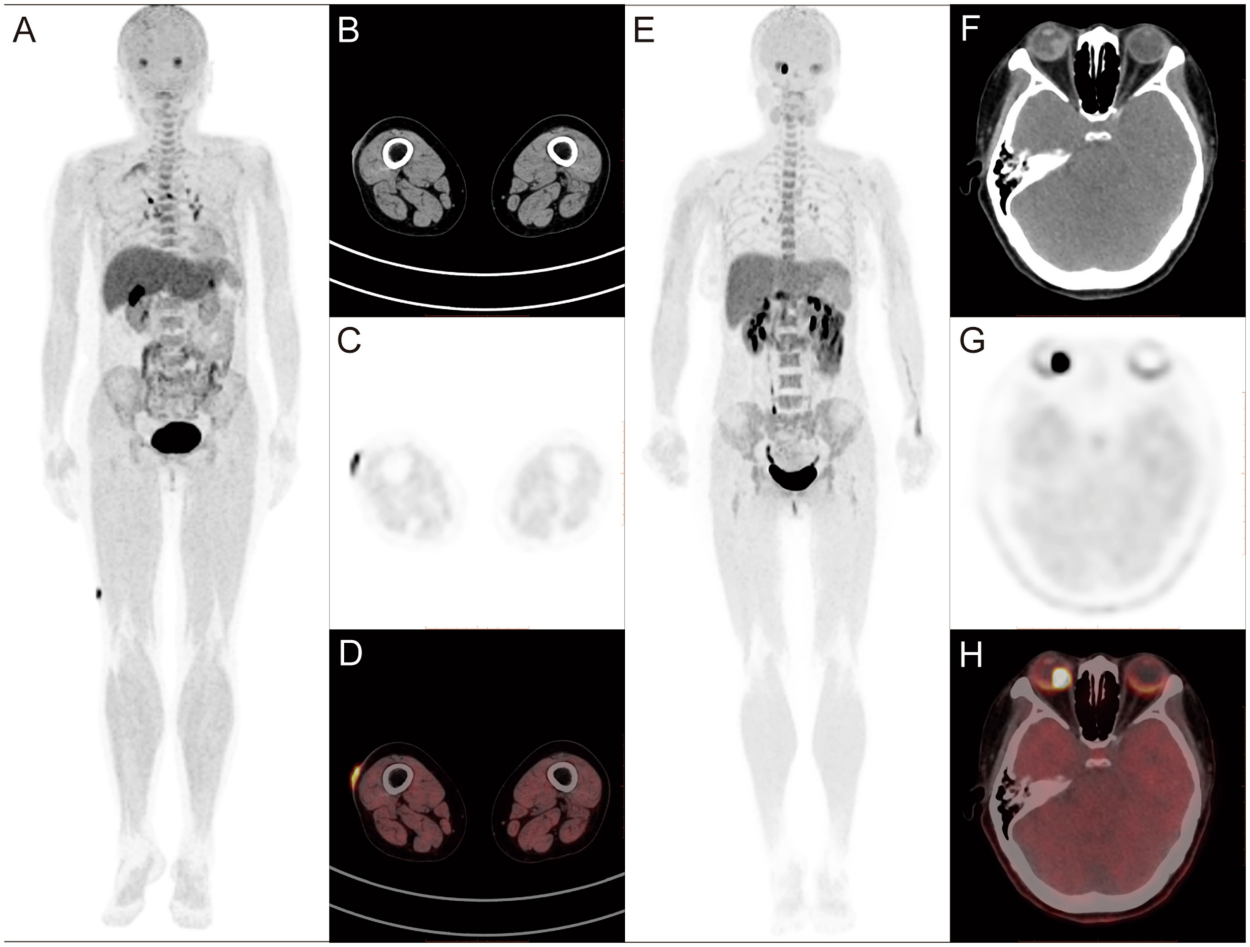
**(A–D)** Findings in a 68-year-old female patient diagnosed with melanoma more than 20 days before the start of the study. ^18^F-P3BZA PET/CT MIP (maximum intensity projection) **(A)** showing a radioactive accumulation shadow on the outer side of the right thigh, and CT **(B)**, PET **(C)**, and PET/CT **(D)** showing local skin thickening on the outer side of the right thigh with increased radioactive uptake (SUVmax: 8.8). **(E–H)** Findings in a 39-year-old female patient diagnosed with melanoma 1 week before the study. ^18^F-P3BZA PET/CT MIP **(E)** showing a radioactive accumulation shadow in the right eye, and CT **(F)**, PET **(G)**, and PET/CT **(H)** showing high-density nodules on the posterior inner wall of the right eyeball, accompanied by radioactive concentration (SUVmax: 33.4).

**Figure 2:**
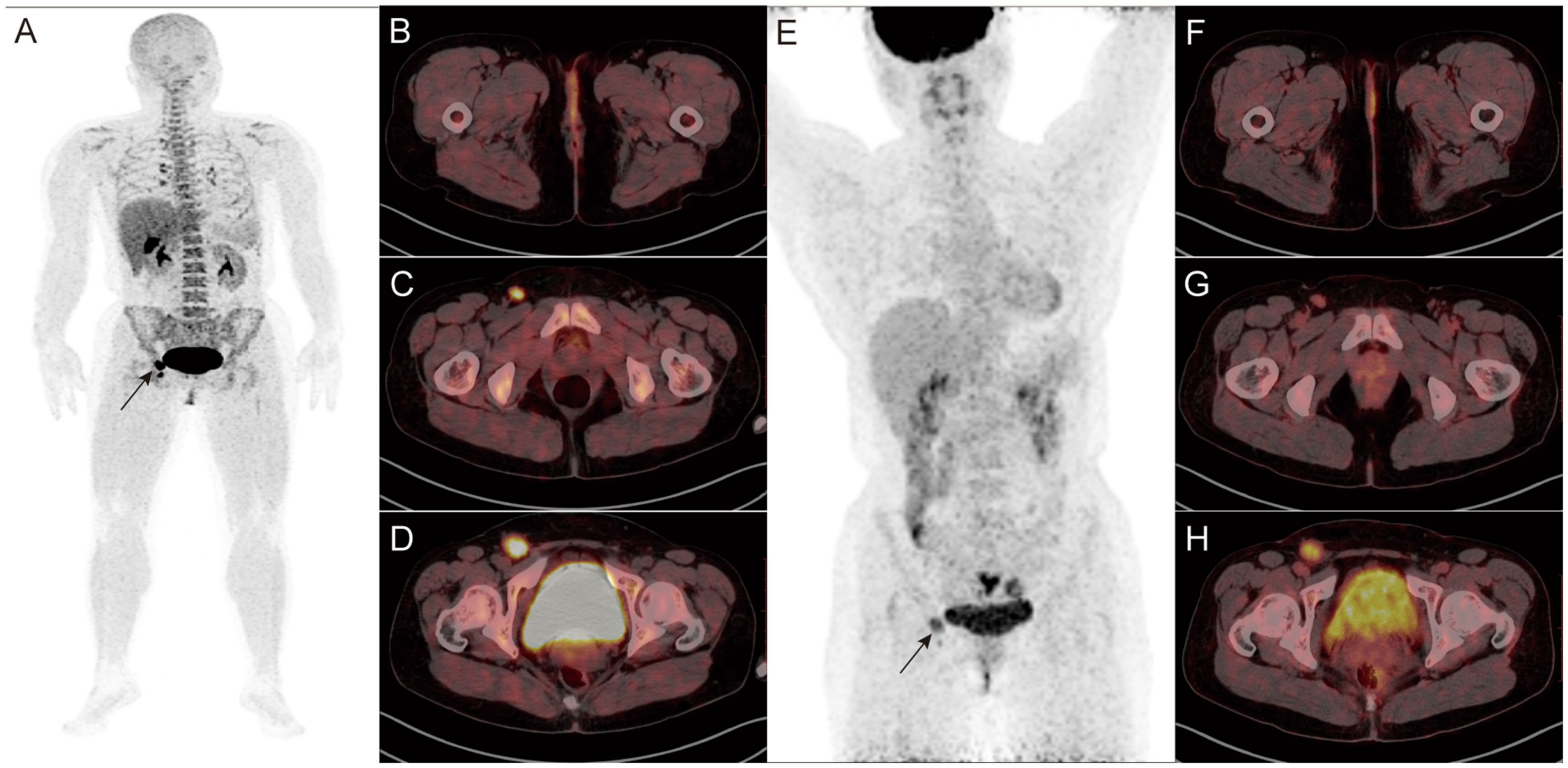
A-H: Findings in a 45-year-old female patient who underwent surgery for external vulvar melanoma 1 year before the start of the study. ^18^F-P3BZA PET/CT MIP **(A)** showing a radioactive accumulation shadow in the right inguinal region (black arrow). Axial PET/CT **(B–D)** showing no significant morphological changes or abnormal uptake in the surgical area, with two enlarged lymph nodes with intense radioactive uptake in the right inguinal region (SUVmax: 7.2 and 14.4, respectively). **(E–H)** ^18^F-FDG PET/CT images of the patient during the same period. ^18^F-FDG PET/CT MIP **(E)** showing a radioactive accumulation shadow in the right inguinal region (black arrow). Axial PET/CT **(F–H)** showing no abnormalities in the surgical area. The radioactive uptake of the two lymph nodes in the right inguinal region is mild to moderate (SUVmax: 3.6 and 5.8, respectively).

^18^F-P3BZA PET/CT detected primary lesions in all newly diagnosed patients (100%, 12/12). The SUVmax of 12 primary tumors was 9.5 ± 9.1, with 10 cases of cutaneous melanoma having an SUVmax of 5.6 ± 3.3 (Figures 1A–D) and 2 cases of ocular melanoma having an SUVmax of 33.4 and 16.8 (Figures 1E–H). No abnormalities were observed at the surgical site in seven postoperative follow-up patients.

As shown in Table 3, ^18^F-P3BZA PET/CT detected 40 metastases. The SUVmax and SUVmean of all metastases were 12.3 ± 10.0 and 6.8 ± 4.1, respectively. The SUVmax of LNM was 11.7 ± 9.4, and that of BM was 8.4 ± 3.0.

**Table 3:** Efficiency analysis of ^18^F-P3BZA PET/CT in the diagnosis of melanoma metastases.

Lesions	Sensitivity	Specificity	PPV	NPV
All metastases	71.4% (40/56)	81.8% (18/22)	90.9% (40/44)	52.9% (18/34)
SLNM	72.4% (21/29)	100% (16/16)	100% (21/21)	61.9% (13/21)
DLNM	66.7% (14/21)	75.0% (12/16)	77.8% (14/18)	63.2% (12/19)
BM	83.3% (5/6)	100% (2/2)	100% (5/5)	66.7% (2/3)

SLNM, sentinel lymph node metastases; DLNM, distant lymph node metastases; BM, bone metastases; PPV, positive predictive value; NPV, negative predictive value.

### ^18^F-FDG PET/CT scan results

Of the 12 newly diagnosed patients, ^18^F-FDG PET/CT detected primary lesions in 11 patients (91.7%, 11/12). No statistically significant difference in sensitivity was observed between ^18^F-FDG and ^18^F-P3BZA (100% vs. 91.7%, *χ*^2^ = 1.043, *p* = 1). The SUVmax of 12 primary tumors was 8.1 ± 5.3, with no statistically significant difference between ^18^F-FDG and ^18^F-P3BZA (*t* = 0.501, *p* = 0.630). In addition, the SUVmax of 10 cases of skin melanoma was 5.6 ± 3.3, and the SUVmax of two cases of ocular melanoma were 3.8 and 8.3. A patient with melanoma of the plantar after surgery presented with false-positive results (mild uptake of ^18^F-FDG).

As shown in Table 4, ^18^F-FDG PET/CT detected 51 metastases. The detection rate of ^18^F-FDG in the diagnosis of DLNM was higher than that of ^18^F-P3BZA (*p* = 0.045). The SUVmax of all metastases was 14.2 ± 7.2. In addition, the SUVmax values for LNM and BM were 15.5 ± 6.4 and 9.3 ± 3.3, respectively. No statistically significant difference in SUVmax was observed between ^18^F-FDG and ^18^F-P3BZA (*t* = 0.877, *p* = 0.410).

**Table 4:** Efficiency analysis of ^18^F-FDG PET/CT in the diagnosis of melanoma metastases.

	Sensitivity	Specificity	PPV	NPV
All metastases	91.2% (51/56)	50.0% (11/22)	80.7% (46/57)	52.3% (11/21)
SLNM	86.2% (25/29)	69.2% (9/13)	86.2% (25/29)	69.2% (9/13)
DLNM	95.2% (20/21)	56.3% (9/16)	74.1% (20/27)	90.0% (9/10)
BM	100% (6/6)	100% (2/2)	100% (6/6)	100% (2/2)

SLNM, sentinel lymph node metastases; DLNM, distant lymph node metastases; BM, bone metastases; PPV, positive predictive value; NPV, negative predictive value.

### Combined imaging scan results

**Figure 3:**
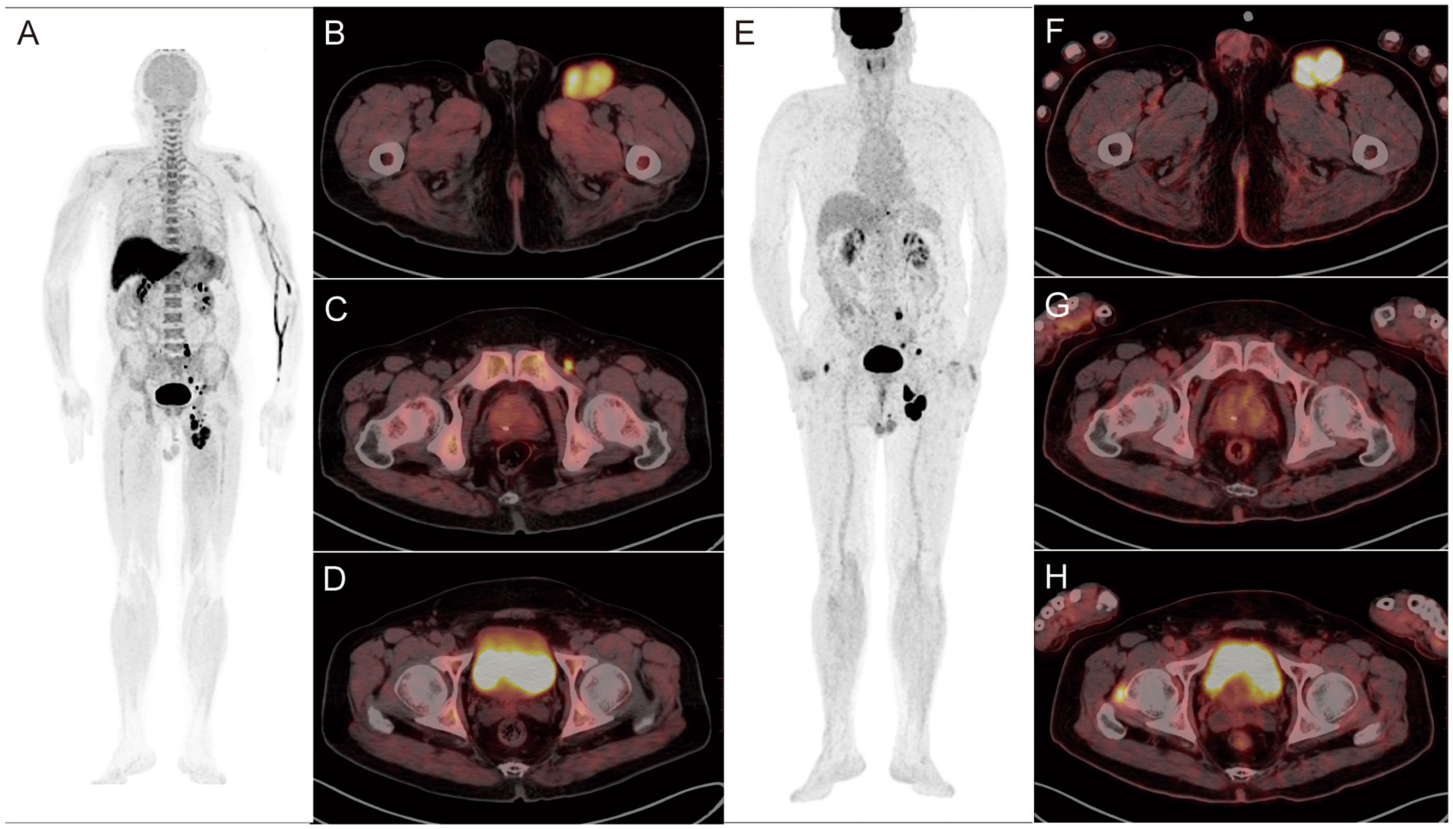
A-H: Findings in a 76-year-old postoperative patient with left plantar melanoma. **(A–D)** ^18^F-P3BZA PET/CT images of the patient. **(E–H)** ^18^F-FDG PET/CT images of the patient. ^18^F-P3BZA PET/CT MIP **(A)** and axial PET/CT **(B,C)** showing multiple enlarged lymph nodes with increased radioactive uptake in the left inguinal region. The positive lymph nodes displayed by ^18^F-FDG PET/CT MIP **(E)** and axial PET/CT **(F,G)** are significantly fewer than those displayed by ^18^F-P3BZA PET/CT. ^18^F-P3BZA PET/CT **(D)** showing no abnormal radioactive uptake in the right femoral head muscle, and ^18^F-FDG PET/CT **(H)** showing a radioactive accumulation shadow, which was confirmed to be a non-melanoma lesion during follow-up.

Combined imaging detected primary lesions in all newly diagnosed patients (100%, 12/12) (Figures 2, 3 A). No statistically significant difference in the detection rate was observed between combined imaging, ^18^F-FDG, and ^18^F-P3BZA (χ^2^ = 2.057, *p* = 0.358).

As shown in Table 5, combined imaging detected 55 metastases. The sensitivity of combined imaging in the diagnosis of SLNM, DLNM, and all metastases was higher than that of ^18^F-P3BZA (*χ*^2^ = 7.105, *p* = 0.004; *χ*^2^ = 3.860, *p* = 0.045; *χ*^2^ = 15.604, *p* < 0.001).

**Table 5:** Efficiency analysis of combined imaging in the diagnosis of melanoma metastases.

	Sensitivity	Specificity	PPV	NPV
All metastases	98.2% (55/56)	100% (22/22)	100% (55/55)	95.7% (22/23)
SLNM	100% (29/29)	100% (13/13)	100% (29/29)	100% (13/13)
DLNM	95.2% (20/21)	100% (16/16)	100% (20/20)	94.1% (16/17)
BM	100% (6/6)	100% (2/2)	100% (6/6)	100% (2/2)

SLNM, sentinel lymph node metastases; DLNM, distant lymph node metastases; BM, bone metastases; PPV, positive predictive value; NPV, negative predictive value.

For all three imaging methods, the diagnostic consistency between two nuclear medicine physicians was good (^18^F-P3BZA: Kappa = 0.762, *p* < 0.001; ^18^F-FDG: Kappa = 0.877, *p* < 0.001; combined imaging: Kappa = 0.825, *p* < 0.001).

**Figure 4:**
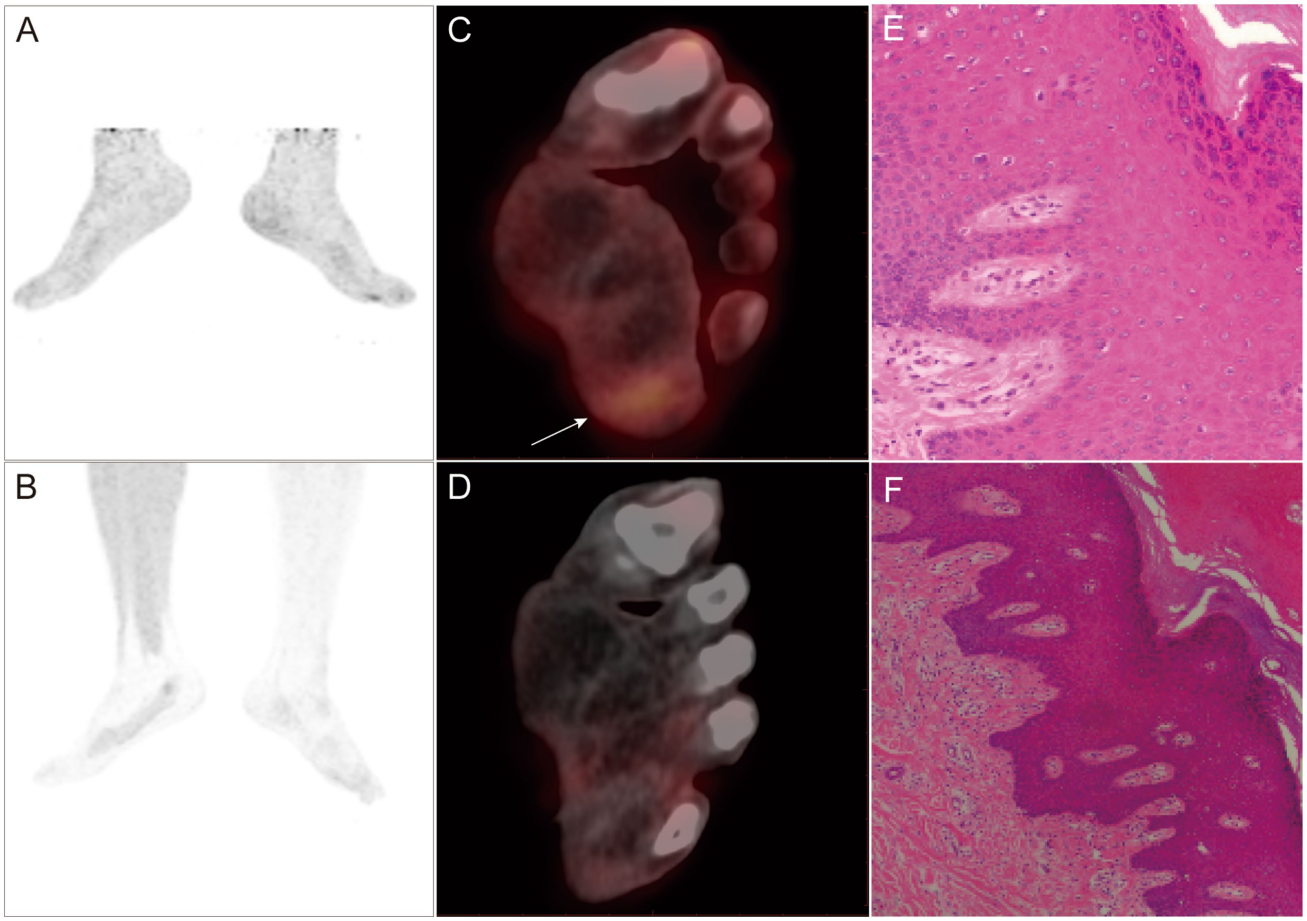
A-F PET/CT and pathological images of a 35-year-old male patient with melanoma who underwent plantar lesion resection surgery 3 weeks before the study took place. ^18^F-FDG PET/CT MIP **(A)** and PET/CT **(C)** showing local radioactive accumulation shadow in the left sole (SUVmax: 2.7). ^18^F-P3BZA PET/CT MIP **(B)** and PET/CT **(D)** without any abnormal radioactive uptake. Five days later, an extended resection of the surgical area was performed, and postoperative pathology confirmed no evidence of tumor recurrence (active local squamous cell proliferation, **E,F**).

Combined imaging improved N and M staging in 31.6% (6/19) of melanoma patients. In addition, it changed the clinical treatment decisions in 26.3% (5/19) of patients (Figure 4; Table 6).

**Table 6:** Combined imaging improves N and M staging results in melanoma.

Imaging method	Patient 3	Patient 6	Patient 8	Patient 11	Patient 12	Patient 17
^18^F-FDG PET/CT	TxN0M1a	TxN2M0	TxN1M0	TxN2M0	TxN2M0	TxN2M0
^18^F-P3BZA PET/CT	TxN0M0	TxN0M0	TxN0M0	TxN0M0	TxN0M0	TxN0M0
Combined imaging	TxN0M1a	TxN2M0	TxN0M0	TxN2M0	TxN0M0	TxN0M0
Confirmed TNM	TxN0M1a	TxN2M0	TxN0M0	TxN2M0	TxN0M0	TxN0M0

## Discussion

The ^18^F-P3BZA used in this study is a derivative of BZA. One hour after injection, ^18^F-P3BZA was mainly distributed in the melanoma lesions of the patients, showing a strong melanin-targeting ability. In addition, the probe showed varying degrees of physiological distribution in the stomach, liver, gallbladder, spleen, and eyes of the patients.

Both the primary lesions and metastases showed a high tumor-to-muscle uptake ratio 60 min after ^18^F-P3BZA injection. The SUVmax of the primary lesion in the eye reached 36.5 ± 19.7. Ma et al. (23) performed ^18^F-P3BZA PET/CT scans on five melanoma patients, and the results showed that the SUVmax values of their primary lesions and metastases were 19.7 ± 5.3 and 18.2 ± 3.7, respectively. Consistent with the results of this study, our findings indicate that ^18^F-P3BZA can specifically bind to melanin *in vivo*, thereby clearly displaying melanoma lesions.

Although ^18^F-P3BZA PET/CT detected all primary lesions (12/12, 100%), its performance in detecting metastases was unsatisfactory, particularly in detecting LNM (70.0%, 35/50), especially DLNM (66.7%, 14/21). This may not be related to the size of the metastases (this study shows that many small LNM also significantly uptake ^18^F-P3BZA) but may be related to the level of melanin in the metastases. In a study on Bomirski hamster melanoma cells, Saud et al. (20) showed that tumor cells exhibit a significantly reduced ability to produce melanin after each generation. Therefore, the insufficient uptake of ^18^F-P3BZA by metastases (especially by DLNM) in the present study may be attributed to a significant decrease in their ability to produce melanin. In addition, we speculate that the synthesis and transport process of melanin requires the participation of multiple enzymes, and the synthesis of these enzymes requires a certain amount of time (27). This leads to a slower increase in melanin levels in metastases (insufficient uptake of ^18^F-P3BZA).

This study showed that the sensitivity, specificity, PPV, and NPV of ^18^F-P3BZA combined with ^18^F-FDG PET/CT in detecting primary lesions were all 100%. Moreover, only one metastasis was missed with the combined imaging, with a sensitivity of 98.2%. It showed significantly lower false-positive and false-negative rates than ^18^F-FDG and ^18^F-P3BZA, respectively. In addition, accurate diagnosis of N and M staging is crucial for the clinical management of patients (28). In the present study, combined imaging improved N and M staging in 31.6% (6/19) of patients, and as a result, treatment plans were changed in 26.3% (5/19) of patients. This change is quite helpful for clinical practice as unnecessary or incorrect treatment can be prevented to some extent. Although there was no statistically significant difference in diagnostic efficacy between combined imaging and ^18^F-FDG alone, the former showed improved final diagnostic results and clinical treatment decisions. However, combined imaging is not suitable for all melanoma patients.

In addition, our PET/CT system is different from the long axial field of view (LAFOV) PET/CT system, which allows for the injection of two tracers on the same day or even simultaneously (29). Although LAFOV PET/CT is currently applied only in a few centers worldwide, this innovative imaging system will soon become available in numerous institutions. Compared to traditional PET/CT systems, LAFOV PET/CT has many advantages in detecting both tumor and non-tumor diseases (30, 31). For instance, using LAFOV, PET/CT PET/CT examinations can be completed on the same day, which can reduce the burden on patients; its higher image quality and optimized lesion quantification can increase lesion detectability; it has low radiation exposure and short collection time; it does not require CT attenuation correction (significantly reducing radiation exposure); and whole-body dynamic imaging can provide more information.

Due to the limitations of using ^18^F-FDG PET/CT for the diagnosis of melanoma in clinical practice, the addition of ^18^F-P3BZA PET/CT as a melanin-specific positron tracer can provide more information for diagnosis. Therefore, this study proposes the following indications for using a combination of ^18^F-FDG and ^18^F-P3BZA PET/CT scanning: (1) the primary lesion or metastasis is located in the brain; (2) conventional ^18^F-FDG PET/CT cannot determine the presence of LNM; (3) there is interference from inflammation in the surgical area; and (4) conventional ^18^F-FDG PET/CT scan is negative although clinical suspicion of melanoma is high.

The findings of this study show that the combination of ^18^F-FDG and ^18^F-P3BZA PET/CT imaging can be effective in diagnosing melanoma, guiding TNM staging, and informing clinical treatment decisions. This can enhance physicians’ confidence in managing melanoma and may indirectly improve patients’ prognosis.

This study has some limitations: (1) the number of patients included in this study is relatively small, and further validation of the findings with a large sample size is needed in the future. In addition, melanoma in the skin and other areas was not been analyzed separately; (2) some patients underwent surgery and medication before the PET/CT scanning, which may have affected the semiquantitative parameters of PET; and (3) this study did not quantitatively analyze melanin in melanoma lesions and investigate its correlation with PET metabolic parameters.

## Data Availability

The original contributions presented in the study are included in the article/supplementary material; further inquiries can be directed to the corresponding authors.
